# Early diagnostic strategy for central nervous system bacterial infections after neurosurgery: A retrospective study

**DOI:** 10.1097/MD.0000000000046635

**Published:** 2025-12-19

**Authors:** Baoli Lin, Xianbing Meng, Ke Pu, Tao Fu, Nanyue Peng, Qingguo Li

**Affiliations:** a Department of Neurosurgery, Huanhu Hospital Affiliated to Tianjin Medical University, Tianjin, China; b Tianjin Key Laboratory of Cerebral Blood Flow Reconstruction and Head and Neck Tumor New Technology Translation, Tianjin, China; c Department of Neurosurgery, The First People’s Hospital of Yulin, Yulin, Guangxi, China; d Department of Neurosurgery, The Second Affiliated Hospital of Shandong First Medical University, Taian, Shandong, China.

**Keywords:** bacterial meningitis, CSF, diagnostic model, mNGS, sensitivity and specificity

## Abstract

Accurate diagnosis of post-neurosurgical bacterial infection of central nervous system is challenging due to the nonspecific nature of clinical signs and cerebrospinal fluid (CSF) parameters, which often overlap with sterile postoperative inflammation. This study aimed to develop and validate a stepwise diagnostic strategy integrating readily available clinical and basic laboratory indicators to improve the early identification of post-neurosurgical bacterial infection of the central nervous system. A retrospective cohort study was conducted at Tianjin Huanhu Hospital, a tertiary neurosurgical center, from October 2018 to June 2025. We enrolled 176 patients suspected of post-neurosurgical bacterial infection of the central nervous system who underwent CSF metagenomic next-generation sequencing (mNGS). Six diagnostic prediction models, combining clinical features (fever, altered mental status) and CSF parameters (white blood cell count, glucose levels), were constructed. Their diagnostic performance was evaluated against a composite reference standard (mNGS, culture, and clinical treatment response) using receiver operating characteristic analysis. The area under the curve (AUC), sensitivity, and specificity were calculated. Among the 6 models, two demonstrated superior performance. Model 5 (T > 38.0°C + [CSF white blood cell ≥ 2000 × 10^6^/L OR CSF glucose < 2.2 mmol/L OR CSF/Blood glucose ratio < 0.4]) achieved an AUC of 0.768. Notably, Model 6 (T > 39.0°C + Altered Mental Status + Intermittent Fever), relying solely on clinical indicators, achieved a comparable AUC of 0.769. For individual parameters, a high fever threshold (T > 39.8°C) and profoundly low CSF glucose (<1.01 mmol/L) showed high specificities of 99%and 97%, respectively, for ruling in infection. A diagnostic strategy combining severe clinical manifestations (high fever and altered mental status) with high-threshold CSF parameters enables effective risk stratification for post-neurosurgical bacterial infection of the central nervous system. The high performance of a purely clinical model (Model 6) offers a valuable tool for rapid bedside assessment, especially in resource-limited settings. Future prospective, multicenter studies are recommended to validate these algorithms and further refine variable definitions for broader clinical application.

## 1. Introduction

The reported incidence of post-neurosurgical bacterial infection (PNBI) of central nervous system (CNS) varies across studies, ranging from 0.3%to 25%.^[1,2]^ With advancements in medical technology and diagnostic practices, the overall incidence of PNBI of CNS is now relatively low. A meta-analysis on post-neurosurgical bacterial meningitis^[3]^ indicated a pooled prevalence of only 2.8% following elective intracranial surgery. In a 14-year investigation into complications after neurosurgical craniotomy, Sajjad et al reported only 1 case of postoperative infection among 305 patients who underwent glioma surgery, and 8 cases among 120 patients undergoing meningioma surgery.^[4]^ Among 1146 patients with cerebellopontine angle tumors, the rates of aseptic and bacterial meningitis were merely 4.54%and 0.87%, respectively. However, severe bacterial meningitis can lead to irreversible neurological damage,^[5,6]^ with mortality rates reaching as high as 55%when caused by multidrug-resistant pathogens.^[7]^ In contrast to the low PNBI of the CNS incidence after elective intracranial surgery,^[3]^ the risk of infection alone following external ventricular or lumbar drainage is considerably higher, reported between 5.5%and 16.2%.^[8,9]^ Therefore, both misdiagnosis of PNBI of CNS – leading to unnecessary drainage procedures and associated iatrogenic infection risks – and underdiagnosis represent significant clinical concerns. To avoid missed diagnoses, neurosurgeons often promptly perform CSF testing or initiate empirical antibiotic treatment when postoperative fever occurs. While necessary for some patients, this approach must be balanced against the fact that postoperative fever is highly common in neurosurgical patients due to both the underlying disease and surgery-induced alterations in CNS metabolism.^[10]^ Subjecting all febrile post-neurosurgical patients to invasive lumbar puncture or even lumbar drainage increases the risk of iatrogenic harm, while misdiagnosis may lead to inappropriate antibiotic use. Nevertheless, early and accurate diagnosis of PNBI of CNS remains a significant clinical challenge. Current reliance on established guidelines is complicated by their inherent limitations. The U.S. Centers for Disease Control and Prevention (CDC) clinical criteria for“Meningitis or Ventriculitis”include”CSF pleocytosis, elevated protein, and/or decreased glucose”but lack specific diagnostic thresholds for these parameters.^[11]^ Similarly, the 2021 Chinese Expert Consensus, while providing specific CSF glucose thresholds (<2.20 mmol/L and a CSF-to-serum ratio ≤ 0.4), recommends clinical and CSF criteria (e.g., fever, intracranial hypertension, and CSF white blood cell (WBC) count > 100 × 10^6^/L) that are common in post-neurosurgical patients and possess limited specificity.^[12]^ This lack of specificity creates a considerable diagnostic dilemma. The clinical presentation of postoperative aseptic meningitis often overlaps significantly with bacterial meningitis, making differentiation based on existing standards challenging and substantially increasing the risk of misdiagnosing sterile inflammation as a bacterial infection.^[13]^ Furthermore, conventional microbiological diagnostics, such as CSF culture, are hampered by inherent drawbacks including prolonged turnaround times and suboptimal sensitivity,^[14,15]^ these reasons created a clear need for improved diagnostic tools. Building on evidence that fever etiology can be discerned through clinical patterns,^[10,16]^ this study systematically evaluated and refined the diagnostic strategy for PNBI of the CNS. Our objective was to develop a practical, data-driven algorithm by defining optimal thresholds for key clinical and laboratory indicators, ultimately aiming to enhance early detection accuracy and guide more targeted clinical management.

## 2. Methods

### 2.1. Study design, setting and period

This retrospective cohort study was conducted at Tianjin Huanhu Hospital, a national-level tertiary specialized center for neurosciences. The study period spanned from October 2018 to June 2025. This extended timeframe was selected to ensure the accumulation of a robust patient cohort evaluated for suspected PNBI of CNS infection, reflecting the real-world application of key diagnostic modalities in this complex clinical setting.

### 2.2. Ethical considerations

The medical ethics committee of of Tianjin Huanhu Hospital approved our research protocol (2025-012). Owing to the retrospective and observational nature of this study, informed consent was not required and was waived. This study adhered to the guidelines outlined in and the standards set by the Declaration of Helsinki. In order to protect the privacy of participants, all personal information was de-identified and anonymized prior to analysis.

### 2.3. Study population

We screened all patients from Department of Neurosurgery, Ward 8, Tianjin Huanhu Hospital for suspected PNBI of the CNS during the study period. All consecutive patients who met the predefined eligibility criteria, as detailed below, were enrolled. No formal sample size calculation was conducted prior to the study. We utilized a consecutive sampling method, whereby all patients admitted during the study period who met the inclusion criteria were enrolled. This approach aimed to maximize statistical power and enhance the representativeness of the sample derived from the source population.

### 2.4. Inclusion criteria

Patients were enrolled if they met all of the following criteria:

1. Underwent one or more invasive neurosurgical procedures with a depth of at least the epidural space within the 30 days preceding cerebrospinal fluid CSF analysis.
2. Developed a clinical suspicion of CNS infection postoperatively, leading to a CSF analysis (including biochemistry, culture, and mNGS) within 30 days after surgery.

### 2.5. Exclusion criteria

Patients were excluded from the analysis for any of the following reasons:

1. Presence of a confirmed intracranial infection prior to surgery, or identification of an active Intracranial infectious focus (e.g., epidural abscess, subdural empyema, brain abscess, or ventricular empyema) during the surgical procedure.
2. History of congenital or acquired immunodeficiency (including but not limited to hereditary immunodeficiency disorders, acquired immunodeficiency syndrome [AIDS], or long-term immunosuppressive therapy).
3. Presence of terminal illness with imminent vital signs failure at the onset of the study, which was deemed unrelated to a potential CNS infection.

### 2.6. Follow-up period

Given the study’s objective to evaluate predictors of infection at the time of CSF testing, a fixed follow-up period for the entire cohort was not applicable. Instead, we defined a patient-specific observation window centered on the key diagnostic event. The follow-up for each enrolled patient spanned a 48-hour period surrounding the CSF sampling for mNGS, encompassing the 24 hours preceding and the 24 hours following the lumbar puncture. This window was chosen to: Capture the clinical presentation (e.g., fever characteristics, Glasgow Coma Scale scores [GCS]) that directly led to the clinical decision to perform the diagnostic tap. Ensure temporal proximity between the predictor variables (including routine CSF biochemistry and cell count from the same sample) and the reference standard. Allow for a clear and contemporaneous correlation between the assessed parameters and the definitive infection status, as determined by the mNGS-based criteria.

### 2.7. Reference standard for diagnosis

To ensure unambiguous outcome classification, a composite reference standard for CNS bacterial infection was established, integrating microbiological results with clinical adjudication. This standard was developed with reference to guidelines from the U.S. Centers for Disease Control and Prevention (CDC) and the Chinese Expert Consensus on the Diagnosis and Treatment of Neurosurgical CNS Infections (2021).

#### 2.7.1. Confirmed positive (infection group):

A patient was classified as having a definitive bacterial infection if they met either of the following criteria:

1. Microbiological confirmation: A positive result from CSF mNGS, irrespective of the CSF culture result.
2. Clinical confirmation with culture: A positive CSF culture, supplemented by a significant clinical and laboratory improvement following the initiation of targeted antimicrobial therapy, even in cases where mNGS was negative.

#### 2.7.2. Confirmed negative (noninfection group):

A patient was classified as not having a bacterial infection if they met either of the following criteria:

1. Negative microbiology with spontaneous resolution: Negative results from both CSF culture and mNGS testing, along with spontaneous resolution of fever and inflammatory indicators in the absence of empirical antibiotic therapy.
2. Ruled-out contamination: A positive result from either CSF culture or mNGS that was deemed to be a contaminant, provided that the patient did not receive targeted antimicrobial therapy against the identified organism and subsequently experienced spontaneous resolution of symptoms and laboratory abnormalities.

### 2.8. Definition of predictors and diagnostic model construction

Key clinical variables were prospectively defined to ensure consistent analysis. Fever and high fever were defined as a body temperature exceeding 38.0°C and 39.0°C, respectively. Based on the temporal profile, fever patterns were categorized into 3 distinct types: Single Fever (an isolated febrile episode), Intermittent fever (recurrent episodes separated by afebrile intervals), and Persistent Fever (a continuous elevation lasting > 6 hours). Guided by this framework and parameter combinations from established guidelines, we constructed 6 distinct diagnostic algorithms (Table 1). These models were designed to systematically assess the diagnostic implications of varying specificity, for instance, by comparing rules that require all laboratory abnormalities versus any one of them. Furthermore, we sought to determine data-driven optimal cutoff points for key continuous variables – specifically, body temperature, CSF white blood cell (WBC) count, and CSF glucose levels. The optimal diagnostic threshold for each variable was identified by maximizing the Youden index (J = sensitivity + specificity-1) derived from ROC curve analysis.

**Table 1:** Definitions of the 5 diagnostic criteria for predicting postoperative central nervous system bacterial infection.

Criterion number	Core components	Detailed definition
1	Fever AND (CSF Pleocytosis OR Hypoglycorrhachia)	- Fever: Body temperature > 38°C - Plus at least one of the following: • CSF WBCt ≥ 100 × 10^6^/L • CSF glucose < 2.20 mmol/L • CSF-to-blood glucose ratio ≤ 0.40
2	Fever AND (Marked CSF Pleocytosis OR Hypoglycorrhachia)	- Fever: Body temperature > 38°C - Plus at least one of the following: • CSF WBC ≥ 1000 × 10^6^/L • CSF glucose < 2.20 mmol/L • CSF-to-blood glucose ratio ≤ 0.40
3	Fever AND CSF Pleocytosis AND Hypoglycorrhachia	- Fever: Body temperature > 38°C - CSF Pleocytosis: WBC ≥ 100 × 10^6^/L - Hypoglycorrhachia: CSF glucose < 2.20 mmol/L or CSF-to-blood glucose ratio ≤ 0.40
4	Fever AND Marked CSF Pleocytosis AND Hypoglycorrhachia	- Fever: Body temperature > 38°C - Marked CSF Pleocytosis: WBC ≥ 1000 × 10^6^/L - Hypoglycorrhachia: CSF glucose < 2.20 mmol/L or CSF-to-blood glucose ratio ≤ 0.40
5	Fever AND Marked CSF Pleocytosis AND Hypoglycorrhachia	- Fever: Body temperature > 38°C - Marked CSF Pleocytosis: WBC ≥ 2000 × 10^6^/L - Hypoglycorrhachia: CSF glucose < 2.20 mmol/L or CSF-to-blood glucose ratio ≤ 0.40
6	Prolonged/High Fever AND Neurological Deterioration	- Fever Pattern (either one): • Prolonged fever: Fever (≥38°C) for > 6 hours per day on 2 consecutive days. • High fever: Single temperature measurement ≥ 39°C. - AND Neurological Deterioration (either one): • GCS decreased persistently by ≥ 1 point. • Mental status persistently worsened.

CSF = cerebrospinal fluid, GCS = Glasgow coma scale, WBC = white blood cell count.

### 2.9. Data source and collection

Data were retrospectively extracted from the electronic medical records of Tianjin Huanhu Hospital. Collected variables encompassed:

Demographics: sex, age, height, weight, and body mass index.
Preoperative&Surgical Data: primary diagnosis, surgical method, and use of prophylactic antibiotics.
Postoperative Clinical Data: body temperature, symptoms and sign (e.g., headache, vomiting, neck stiffness), therapeutic antibiotic use, GCS scores, and documented alterations in mental status.
Laboratory Data: CSF parameters (collection time, routine biochemistry, culture, and mNGS results performed by Tianjin Mailluo Medical Laboratory Co., Ltd.).

To ensure temporal relevance, data were aligned with a key diagnostic event. For patients with multiple tests, the first CSF sample confirming infection (or the first sample after cessation of invasive procedures for noninfected patients) was selected. Clinical data – including the worst GCS score and the presence of altered mental status – were strictly referenced to the 24-hour period preceding this definitive lumbar puncture. All data were systematically compiled into a predefined electronic case report form for analysis.

**Figure 1.**
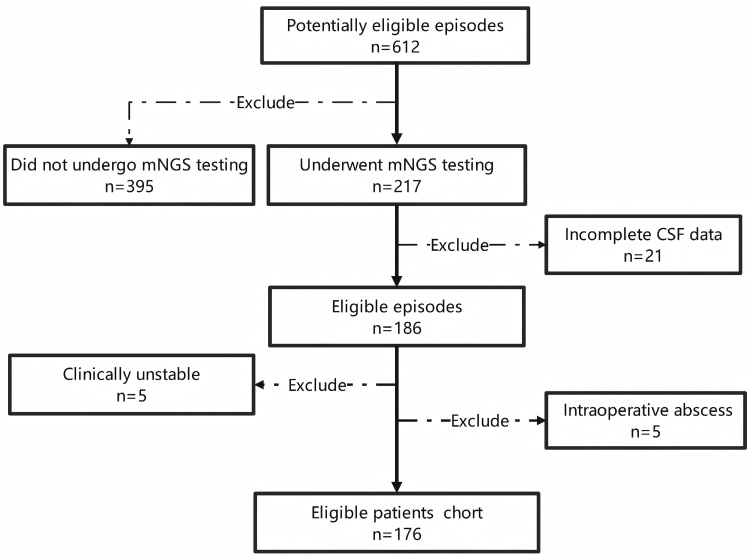
Flow chart of patient recruitment. This diagram illustrates the process of identifying eligible patients in the retrospective cohort study, including the application of inclusion and exclusion criteria. CSF = cerebrospinal fluid.

Patients with incomplete key data (e.g., CSF biochemistry) were excluded from the final analysis, as shown in Figure 1.

### 2.10. Statistical analysis

Definition of Study Groups for Comparison: The primary comparative analysis was performed between the following 2 groups, defined by the reference standard described in section“Reference Standard for Diagnosis”

Group 1 (infection): Patients with a confirmed diagnosis of PNBI of CNS.
Group 2 (noninfection): Patients who did not meet the criteria for confirmed diagnosis of PNBI of CNS.

All statistical analyses were performed using R software (version 4.2.2, R Foundation for Statistical Computing, Vienna, Austria), along with the use of MSTATA software (https://www.mstata.com/). The normality of continuous data was assessed using the Shapiro–Wilk test, normally distributed continuous data are presented as mean ± standard deviation (Mean ± SD) and were compared between groups using the independent samples t-test. Non-normally distributed continuous data are expressed as median and interquartile range [M(Q1, Q3)], and comparisons were performed using the Mann–Whitney U test. Categorical data are presented as counts and proportions[n(%)]. Unordered categorical data were compared between groups using Pearson chi-square test or Fisher exact test, as appropriate, while ordered categorical data were compared using the Mann–Whitney U test. To further evaluate the balance between groups, standardized mean differences (SMD) were reported. Generally, an SMD < 0.10 indicates excellent balance; 0.10 to 0.34 suggests a small difference; 0.35 to 0.64 indicates a moderate difference; 0.65 to 1.19 represents a substantial difference; and an SMD ≥ 1.20 indicates a very large difference between groups.

The baseline table of demographic and clinical characteristics was generated using the“Descriptive Statistics”module of the MSTATA statistical platform. The diagnostic performance of the 6 predictive criteria and individual continuous variables (e.g., CSF WBC count, glucose levels) was rigorously evaluated. For each predictor, a receiver operating characteristic (ROC) curve was constructed, and the area under the curve (AUC) along with its 95% confidence interval was calculated to quantify overall discriminative ability. To derive binary classification metrics – including sensitivity, specificity, positive predictive value (PPV), and negative predictive value – for the 6 diagnostic criteria, patients were classified as positive or negative according to each criterion’s predefined rules. These classifications were then cross-tabulated against the reference standard (the final diagnosis of CNS bacterial infection). Sensitivity, specificity, PPV, and negative predictive value, together with their 95% confidence intervals, were computed directly from the resulting 2 × 2 contingency tables. For continuous variables, the optimal cutoff value for predicting infection was identified by maximizing the Youden index (J = sensitivity + specificity-1). All analyses were performed using the”Diagnostic Study”module of the MSTATA statistical platform.

### 2.11. Clinical care protocol

The standard management of postoperative fever in our neurosurgical ward follows a tiered protocol. Body temperature is monitored at 4-hour intervals for stable patients, increasing to 1 to 2 hours for febrile episodes (≥38.0°C). Persistent fever triggers a diagnostic workup, including blood and urine tests, chest imaging, and inflammatory markers. When intracranial infection is suspected, cerebrospinal fluid is obtained for cell count, biochemistry, culture, and mNGSas clinically indicated.

## 3. Results

### 3.1. Participant selection and population baseline characteristics

A total of 612 patients suspected of having PNBI were screened. Among them, 416 patients were excluded: 395 due to the absence of cerebrospinal fluid (CSF) mNGS testing, while the remaining 217 patients underwent CSF mNGS testing, of whom 21 were excluded due to incomplete CSF data about routine biochemistry. Ultimately, 186 patients who had undergone neurosurgery met the primary inclusion criteria. After further review based on exclusion criteria, 5 patients with life-threatening conditions unrelated to infection at the start of the study and 5 patients with intraoperatively confirmed Intracranial abscess were excluded. Finally, 176 eligible patients were included in the study (Fig. 1).

Our assessment of selection bias revealed that the 217 included and 395 excluded patients were well-balanced, with only minimal differences (SMD < 0.3) in a few variables (Table 2). In the final cohort, the 76 patients with CNS infection and 100 noninfected controls were similar in all key demographic and surgical metrics. Key differences emerged in antibiotic exposure, which was significantly more frequent in the infection group both before surgery and prior to CSF collection (Table 3).

**Table 2:** Comparison of baseline characteristics between included patients and those excluded for not undergoing mNGS testing.

Characteristic	mNGS		*P*-value	SMD
	No N = 395	Yes N = 217		
Male sex, n (%)	218 (55.2%)	142 (65.4%)	.014*	0.211
Age (years), Median (Q1, Q3)	55 (41, 64)	52 (36, 62)	.019†	0.244
Height (cm), Median (Q1, Q3)	170 (160, 173)	170 (162, 174)	.064†	0.035
Body weight (kg), Median (Q1, Q3)	68 (61, 76)	67 (64, 78)	.196†	0.105
BMI, Median (Q1, Q3)	23.7 (21.7, 26.5)	24.5 (21.8, 26.8)	.334†	0.062
Diabetes, n (%)	57 (14.4%)	12 (5.5%)	<.001*	0.300
CSF-Surgery Interval, Median (Q1, Q3)	7 (4, 11)	8 (5, 14)	.057†	0.187
Intracranial prior surgery, n (%)	43 (10.9%)	34 (15.7%)	.088*	0.141
Preoperative diagnosis, n (%)				
Aneurysm	41 (10.4%)	15 (6.9%)	.200*	0.218
Brain injury	13 (3.3%)	2 (0.9%)		
Hydrocrania	22 (5.6%)	11 (5.1%)		
HCH	102 (25.8%)	64 (29.5%)		
Intracranial Mass Lesion	217 (54.9%)	125 (57.6%)		
Operative method, n (%)				
Burr Hole	99 (25.1%)	58 (26.7%)	.652*	0.038
craniotomy	296 (74.9%)	159 (73.3%)		
Operative approach, n (%)				
Cranial Base approach	123 (31.1%)	75 (34.6%)	.360*	0.123
Transcortical approach	228 (57.7%)	125 (57.6%)		
Transsphenoidal approach	44 (11.1%)	17 (7.8%)		
Preoperative prophylactic antibiotics, n (%)	173 (43.8%)	69 (31.8%)	.004*	0.249
Dura repared, n (%)	283 (71.6%)	144 (66.4%)	.173*	0.114
V-P shunt, n (%)	19 (4.8%)	3 (1.4%)	.029*	0.199

A comparison of baseline characteristics between the included and excluded cohorts revealed good overall balance, with all SMD below the threshold of 0.3.

BMI = body mass index, CSF = cerebrospinal fluid, HCH = hypertensive cerebral hemorrhage, SMD = standardized mean difference, V-P = ventriculoperitoneal.

* Pearson’s Chi-squared test.

† Wilcoxon rank sum test

**Table 3:** Comparison of baseline characteristics between patients with and without central nervous system infection following neurosurgery in ward 8, Tianjin Huanhu Hospital (2018–2025).

Characteristic	Infection		*P*-value	SMD
	No N = 100	Yes N = 76		
Male sex, n (%)	62 (62.0%)	50 (65.8%)	.605*	0.079
Age (years), Mean ± SD	48 ± 19	48 ± 20	.980†	0.004
Height (cm), Median ± SD	167.8 ± 16.0	164.7 ± 16.3	.214	0.191
Body weight (kg), Median (Q1, Q3)	73.5 (62.0, 80.0)	69.5 (63.0, 78.3)	.457	0.169
BMI, Mean ± SD	26.9 ± 23.0	24.5 ± 3.9	.316†	0.144
Preoperative diagnosis, n (%)				
Aneurysm	12 (12.0%)	4 (5.3%)	.218‡	0.364
Brain injury	1 (1.0%)	3 (3.9%)		
Hydrocrania	1 (1.0%)	2 (2.6%)		
HCH	24 (24.0%)	24 (31.6%)		
Intracranial Mass Lesion	62 (62.0%)	43 (56.6%)		
Diagnosed with Diabetes, n (%)	6 (6.0%)	8 (10.5%)	.272*	0.165
History of intracranial surgery, n (%)	9 (9.0%)	12 (15.8%)	.169*	0.207
Operative method: craniotomy, n (%)	81 (81.0%)	56 (73.7%)	.247*	0.175
Operative approach, n (%)				
Cranium approach	55 (55.0%)	42 (55.3%)	.072*	0.348
Skull base approach	38 (38.0%)	21 (27.6%)		
Sphenoid sinus approach	7 (7.0%)	13 (17.1%)		
Preoperative prophylactic Antibiotics, n (%)	24 (24.0%)	36 (47.4%)	.001*	0.503
Antibiotics administered prior to CSF collection, n (%)	36 (36.0%)	47 (61.8%)	<.001*	0.535

There were no missing data for any variable presented in this table.

This table compares the demographic and clinical profiles of 76 patients who developed a central nervous system (CNS) infection and 100 noninfected controls. Categorical data are presented as n (%). Continuous data are presented as Mean ± SD or Median (Q1, Q3). *P*-values were derived from the Welch *t*-test^1^, Pearson Chi-squared test^2^, or Fisher exact test^3^. Standardized Mean Differences (SMD) are also provided. The 2 groups were well-balanced in all key baseline characteristics (SMD mostly <0.3), except for significantly higher rates of antibiotic exposure in the infection group, both before surgery and prior to CSF collection.

BMI = body mass index, CSF = cerebrospinal fluid, HCH = Hypertensive Cerebral Hemorrhage.

* Pearson’s Chi-squared test.

† Welch two sample *t*-test.

‡ Fisher’s exact test.

### 3.2. CSF pathogen detection: culture versus mNGS

**Figure 2.**
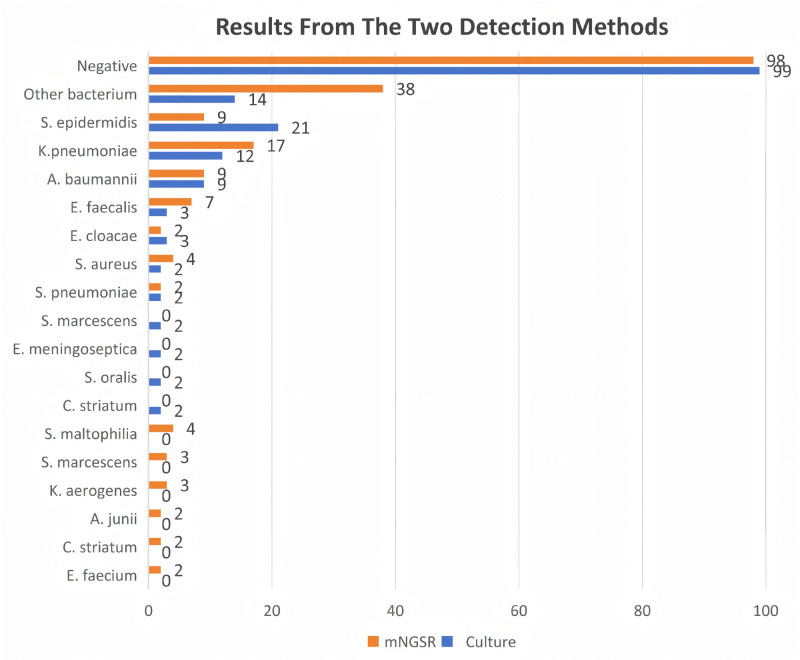
Comparison of pathogen detection by CSF Culture with mNGS. In the 76 patients with confirmed central nervous system bacterial infection, mNGS demonstrated a higher diagnostic yield than conventional culture. mNGS identified a broader spectrum of pathogens, including fastidious organisms missed by culture. Discordant results were observed in 31 cases, with 18 instances positive only by mNGS and 13 positive only by culture. S. marcescens: Serratia marcescens; S. oralis: Streptococcus oralis; A. baumannii: Acinetobacter baumannii; C. striatum: Corynebacterium striatum; E. faecalis: Enterococcus faecalis; K. aerogenes: Klebsiela aerogenes; S. aureus: Staphylococcus aureus; S. maltophilia: Stenotrophomonas maltophilia; S. pneumoniae: Streptococcus pneumonia. CSF = cerebrospinal fluid, mNGS = metagenomic next-generation sequencing.

**Figure 3.**
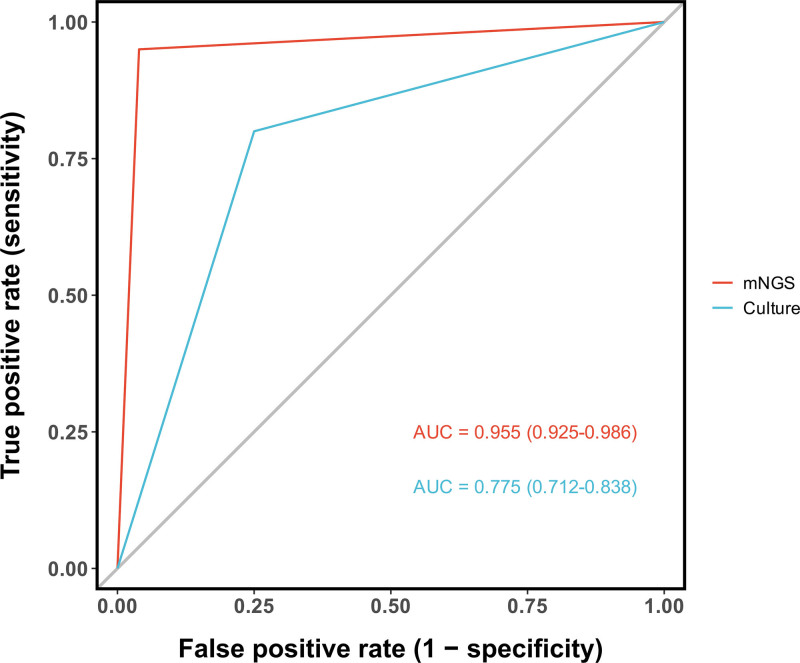
Diagnostic accuracy of mNGS versus culture. The ROC analysis shows mNGS has excellent diagnostic accuracy (AUC = 0.955) for CNS bacterial infection, which is markedly higher than that of conventional culture (AUC = 0.775). CNS = central nervous system, mNGS = metagenomic next-generation sequencing, ROC = receiver operating characteristic.

A direct comparison of CSF conventional culture with mNGS was performed in the 176-patient cohort. As detailed in Figure 2, mNGS demonstrated a markedly higher detection rate for most pathogens. For instance, it identified 3 times as many Staphylococcus aureus cases (12 vs 4 by culture) and more than doubled the detection of Stenotrophomonas maltophilia (9 vs 4) and Enterococcus faecalis (7 vs 3). Consequently, a substantially larger number of samples were positive exclusively by mNGS (n = 38) than exclusively by culture (n = 14). Assessment against the final composite clinical reference standard confirmed the superior diagnostic accuracy of mNGS over culture (Fig. 3). This advantage was most evident in the subgroup of 76 patients with a confirmed CNS bacterial infection, where mNGS provided a markedly higher diagnostic yield.

### 3.3. Diagnostic predictive capabilities

#### 3.3.1. Fever patterns:

**Figure 4.**
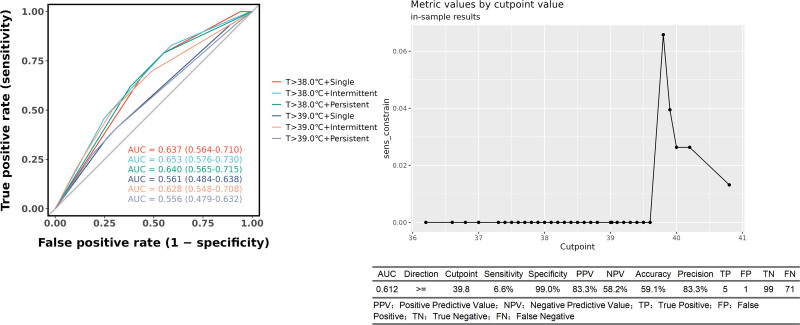
Diagnostic performance of different fever patterns for CNS bacterial infection. (A) ROC analysis of 6 fever patterns shows limited and comparable discriminative ability, with all AUC values below 0.66. The intermittent fever pattern at a threshold of >38.0°C demonstrated a marginally higher AUC, while persistent fever at >39.0°C had the lowest. (B) Although no pattern was highly accurate, a single temperature above 39.8°C achieved a specificity of 99%, offering high rule-in value. CNS = central nervous system.

The diagnostic performance of various fever patterns, as defined in the Methods section, was evaluated using ROC analysis (Fig. 4). Among the 6 fever patterns analyzed, the AUC values were as follows:T > 38.0°C + Single:AUC = 0..637 (95%CI: 0.564–0.710), T > 38.0°C + Intermittent:AUC = 0..653 (95%CI: 0.576–0.730), T > 38.0°C + Persistent:AUC = 0..640 (95%CI: 0.565–0.715), T > 39.0°C + Single:AUC = 0..561 (95%CI: 0.484–0.638), T > 39.0°C + Intermittent:AUC = 0..628 (95%CI: 0.548–0.708), T > 39.0°C + Persistent:AUC = 0..556 (95%CI: 0.479–0.632), The intermittent fever pattern at T > 38.0°C showed a marginally higher AUC than other patterns, though the differences were not statistically significant. In contrast, the persistent high fever pattern (T > 39.0°C + Persistent) demonstrated the lowest discriminative ability. Analysis of temperature thresholds revealed: Using T > 38.0°C yielded a sensitivity of 0.83 and specificity of 0.33. A lower threshold of T > 37.5°C increased sensitivity to 0.90 but specificity fell to 0.11. A high threshold of T > 39.8°C provided a specificity of 0.99, indicating high rule-in potential for bacterial CNS infection when present.

#### 3.3.2. Clinical symptoms and signs:

**Figure 5.**
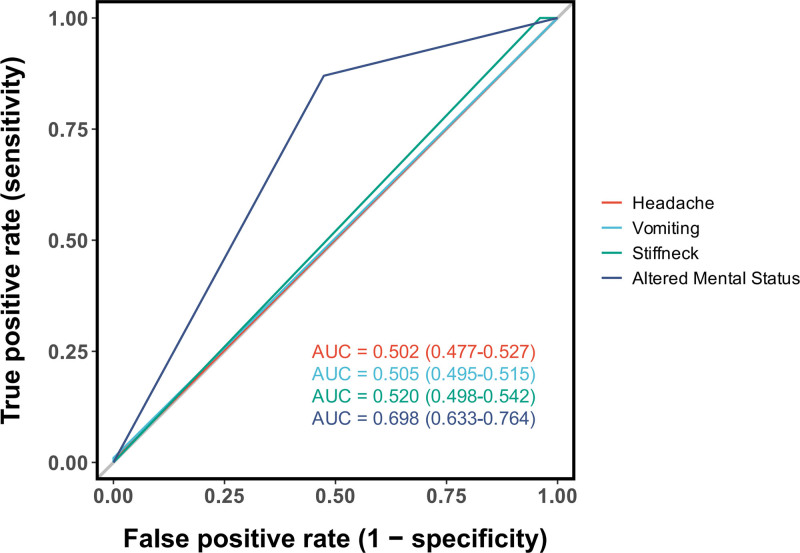
Diagnostic performance of clinical signs. Among the signs evaluated, only altered mental status provided substantial diagnostic value (AUC = 0.698) for identifying CNS bacterial infection. CNS = central nervous system.

The diagnostic utility of key clinical signs was evaluated using ROC analysis (Fig. 5). Among the variables assessed, altered mental status – defined as a decrease in GCS score or slower reactions – was the only substantial predictor, with an AUC of 0.698 (95%CI: 0.633–0.764). In contrast, classic meningeal signs such as headache (AUC = 0.502), vomiting (AUC = 0.505), and stiff neck (AUC = 0.520) demonstrated discriminative ability no better than chance in this cohort (Fig. 5). A subgroup analysis of the 115 febrile patients (body temperature > 38.0°C) revealed that 39 (33.9%) presented with altered mental status (Table 4). The 2 groups – with and without altered mental status – were comparable in terms of sex, age, body mass index, and prevalence of diabetes (all *P* > .05). However, significant differences were observed in underlying diagnoses and clinical management. Patients with altered mental status had a significantly higher proportion of hypertensive cerebral hemorrhage as the preoperative diagnosis (41.0% vs 26.3%), while none were diagnosed with aneurysm. Regarding surgical interventions, external ventricular drainage was more frequently performed in the altered mental status group (33.3% vs 15.8%, *P* = .031). Additionally, this group had a significantly higher rate of preoperative prophylactic antibiotic use (46.2% vs 21.1%, *P* = .005). A marked contrast was also observed in the use of drainage devices (*P* < .001): while most patients without altered mental status did not require drainage (80.3%), nearly half of those with altered mental status (46.2%) received lumbar cisternal drainage.

**Table 4:** Baseline characteristics by altered mental status in febrile patients (T >38.0°C).

Characteristic	T > 38.0°C (yes), N = 115		
	Altered mental status (no) N = 76	Altered mental status (yes) N = 39	*P*-value*
Male sex, n (%)	45 (59.2%)	27 (69.2%)	.293
Age (year), Median (Q1, Q3)	52 (37, 62)	49 (33, 58)	.460
Height (cm), Median (Q1, Q3)	169 (163, 176)	170 (163, 173)	.896
Body weight (kg), Median (Q1, Q3)	71 (63, 79)	68 (63, 79)	.965
BMI, Median (Q1, Q3)	24.9 (22.7, 26.8)	24.7 (22.6, 26.6)	.976
Diabetes, n (%)	3 (3.9%)	4 (10.3%)	.225
Preoperative diagnosis, n (%)			
Aneurysm	10 (13.2%)	0 (0.0%)	.019
Brain injury	2 (2.6%)	2 (5.1%)	
Hydrocrania	0 (0.0%)	1 (2.6%)	
HCH	20 (26.3%)	16 (41.0%)	
Intracranial mass lesion	44 (57.9%)	20 (51.3%)	
Operative method, n (%)			
Burr hole	12 (15.8%)	13 (33.3%)	.031
craniotomy	64 (84.2%)	26 (66.7%)	
Preoperative prophylactic antibiotics, n (%)	16 (21.1%)	18 (46.2%)	.005
Antibiotics administered prior to CSF collection, n (%)	30 (39.5%)	22 (56.4%)	.084
Drainage tube, n (%)			
EVD	5 (6.6%)	4 (10.3%)	<.001
ELD	10 (13.2%)	18 (46.2%)	
None	61 (80.3%)	17 (43.6%)	

In Febrile Patients (T >38.0°C): Patients with altered mental status differed significantly from those without in their preoperative diagnosis, operative method, and clinical management. They had a higher utilization of preoperative prophylactic antibiotics and LCD, suggesting these factors are potential confounders. In Non-Febrile Patients (T ≤ 38.0°C): The groups with and without altered mental status were largely comparable in most baseline characteristics, with the only significant differences observed in preoperative and pre-CSF collection antibiotic exposure.

CSF = cerebrospinal flu, ELD = external lumbar drainage, EVD = external ventricular drainage, HCH = hypertensive cerebral haemorrhage.

* Fisher’s exact test; Wilcoxon rank sum test; Pearson Chi-squared test.

#### 3.3.3. CSF cell count:

**Figure 6.**
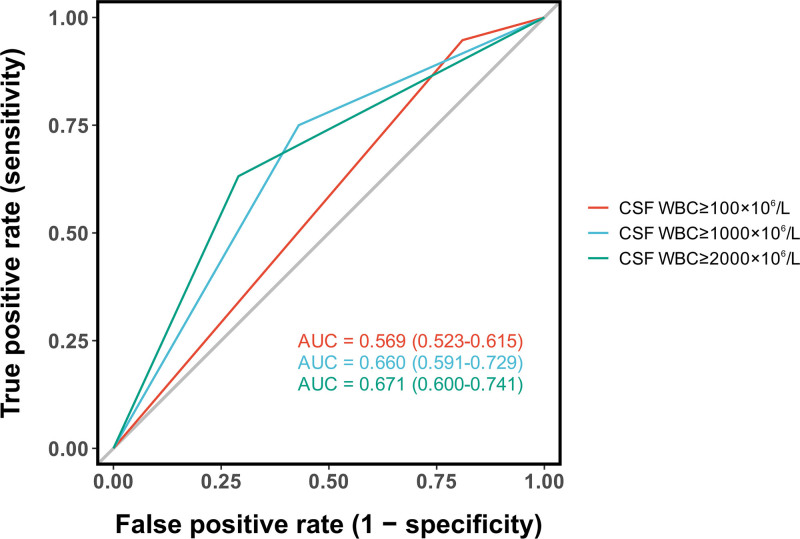
Diagnostic performance of CSF WBC count thresholds. ROCs demonstrate that increasing the CSF WBC count threshold improves the ability to discriminate between bacterial infection and postoperative inflammation. The AUC increases from 0.569 at ≥ 100 × 10^6^/L to 0.671 at ≥ 2000 × 10^6^/L. AUC = area under the curve, CSF = cerebrospinal fluid, ROC = receiver operating characteristic, WBC = whit blood cell.

**Figure 7.**
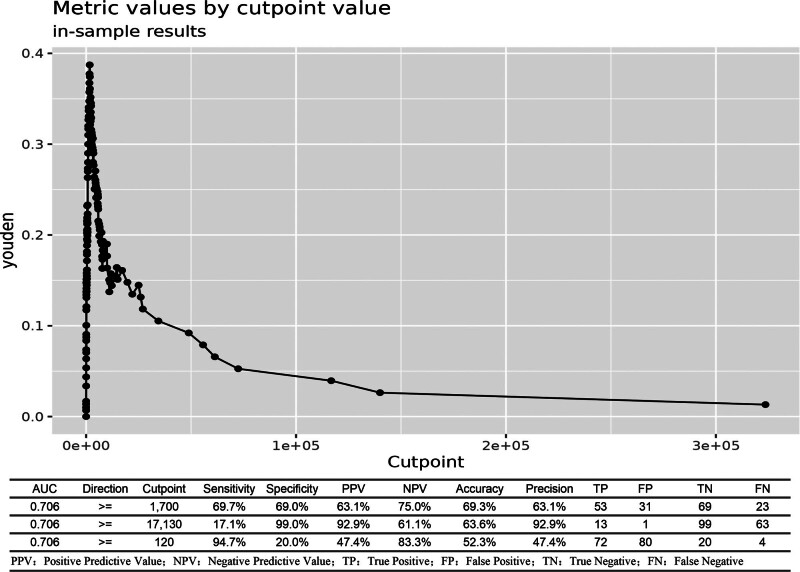
Optimal diagnostic threshold for CSF WBC count. The ROC analysis of CSF WBC count as a continuous variable yielded an AUC of 0.706. The Youden index identified 1700 × 10^6^/L as the optimal cutoff, providing a balance between sensitivity (69.7%) and specificity (69.0%). The analysis also illustrates the trade-off at extreme thresholds: a very low cutoff (120 × 10^6^/L) maximizes sensitivity (94.7%) but sacrifices specificity, while a very high cutoff (17,130 × 10^6^/L) achieves near-perfect specificity (99%) at the cost of low sensitivity. AUC = area under the curve, CSF = cerebrospinal fluid, ROC = receiver operating characteristic, WBC = whit blood cell.

The diagnostic performance of various CSF WBC count thresholds was systematically evaluated. ROC curve comparison of 3 different cutoffs revealed a progressive improvement in discriminatory power with increasing WBC thresholds: the AUC for CSF WBC ≥ 100 × 10^6^/L was 0.569 (95%CI: 0.523–0.615), which increased to 0.660 (95%CI: 0.591–0.729) for ≥ 1000 × 10^6^/L, and further to 0.671 (95%CI: 0.600–0.741) for ≥ 2000 × 10^6^/L (Fig. 6). Analysis of the sensitivity-specificity trade-off at these thresholds showed: At a lower threshold of > 120 × 10^6^/L, sensitivity reached 94.7%, while specificity was only 20%. At a high threshold of > 17,130 × 10^6^/L, specificity was as high as 99%, but sensitivity dropped markedly to 17%. The overall ROC analysis for CSF WBC count as a continuous variable yielded an AUC of 0.706 (95%CI: 0.628–0.784), with an optimal cutoff of 1700 × 10^6^/L as determined by the Youden index (Fig. 7).

### 3.4. Diagnostic value of CSF glucose and CSF-to-blood glucose ratio

**Figure 8.**
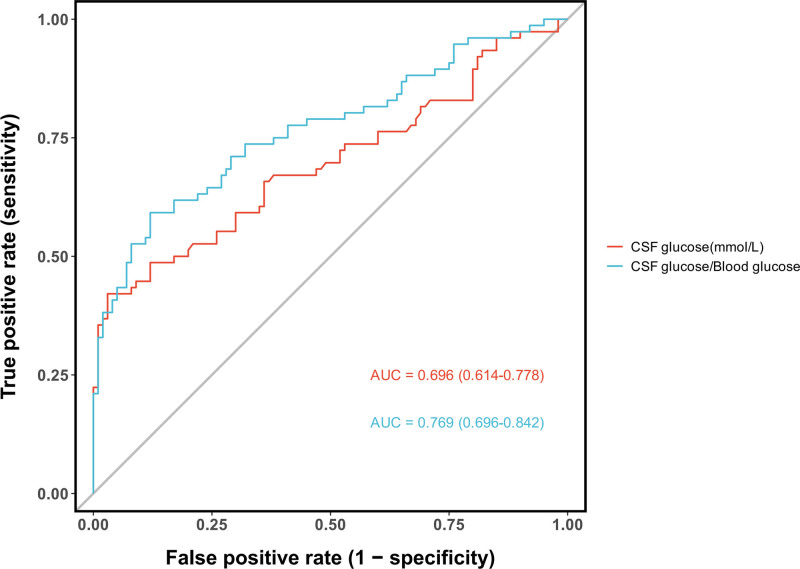
Diagnostic performance of CSF glucose and CSF-to-blood glucose ratio. ROC analysis demonstrates that the CSF-to-blood glucose ratio (AUC = 0.769) is a superior discriminator for central nervous system bacterial infection compared to absolute CSF glucose levels alone (AUC = 0.696). AUC = area under the curve, CSF = cerebrospinal fluid, ROC = receiver operating characteristic.

**Figure 9.**
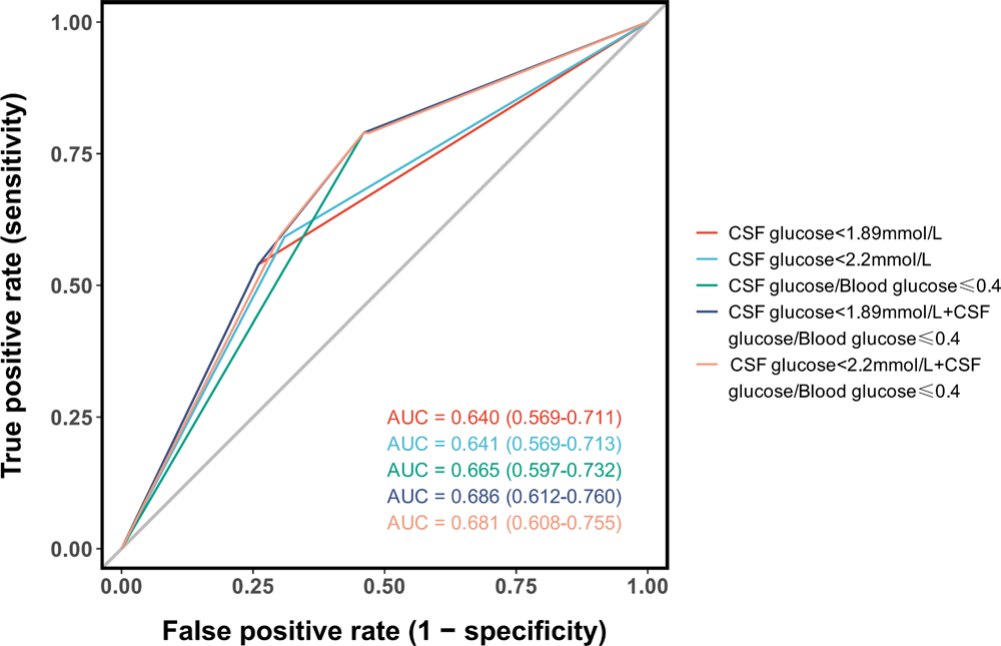
Comparison of diagnostic accuracy for different glucose-related indicators. The combination of a low CSF glucose level (using either < 2.20 mmol/L or < 1.89 mmol/L threshold) with a low CSF-to-blood glucose ratio (≤0.4) achieved the highest diagnostic accuracy (AUC = 0.686), outperforming any of these individual parameters used alone. AUC = area under the curve, CSF = cerebrospinal fluid.

**Figure 10.**
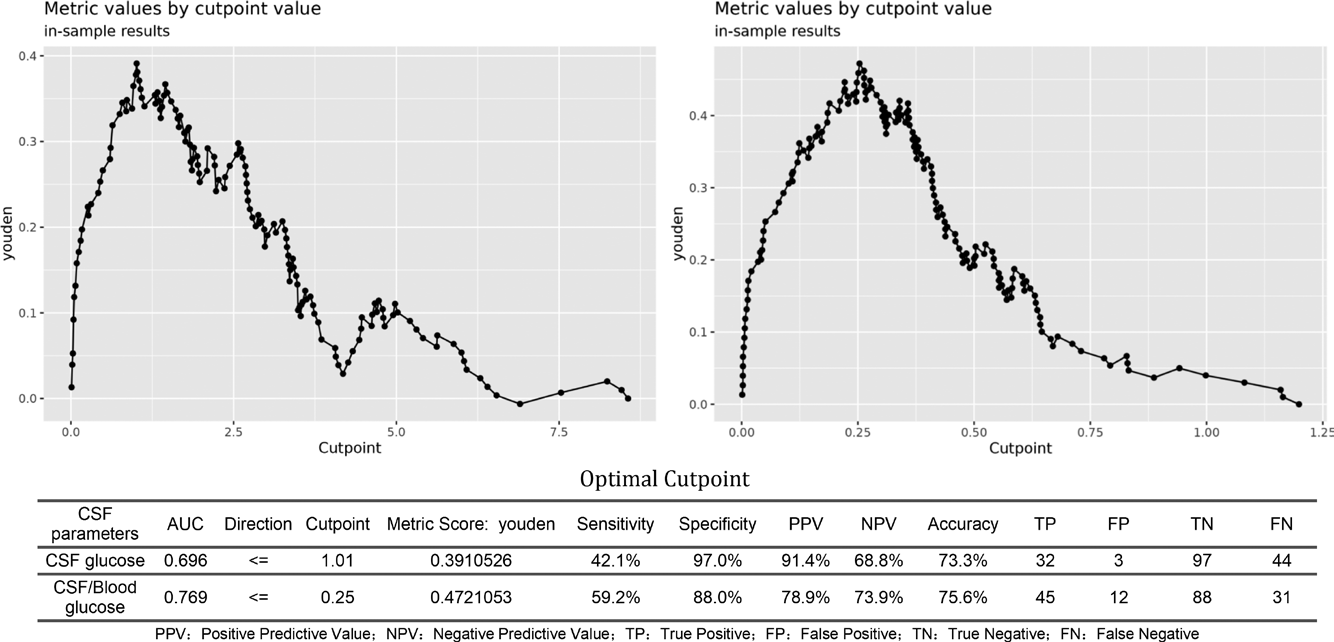
Optimal diagnostic thresholds for CSF glucose parameters. Analysis identified a CSF glucose level of ≤1.01 mmol/L as a highly specific (97.0%) indicator for CNS bacterial infection, with a high positive predictive value (91.4%). The CSF-to-blood glucose ratio demonstrated superior overall performance (AUC = 0.769), with an optimal cutoff of ≤0.25 providing a more balanced sensitivity (59.2%) and specificity (88.0%). AUC = area under the curve, CNS = central nervous system, CSF = cerebrospinal fluid.

The diagnostic utility of CSF glucose parameters was systematically evaluated using ROC analysis. The CSF-to-blood glucose ratio demonstrated superior discriminative ability (AUC = 0.769,95%CI: 0.696–0.842) compared to the absolute CSF glucose level alone (AUC = 0.696,95%CI: 0.614–0.778)(Fig. 8). A comparison of various glucose-related diagnostic indicators further revealed that combined criteria outperformed any single parameter. Specifically, the combination of a low CSF glucose level (using either the conventional < 1.89 mmol/L or the < 2.2 mmol/L threshold) with a low CSF-to-blood glucose ratio (≤0.4) achieved the highest diagnostic accuracy, with AUCs of 0.686 and 0.681, respectively. These combined models provided a better balance than using low CSF glucose (AUC = 0.640–0.641) or a low glucose ratio (AUC = 0.665) in isolation (Fig. 9). Analysis of optimal diagnostic thresholds identified a CSF glucose level of ≤ 1.01 mmol/L as a highly specific marker (97.0%), with a PPV of 91.4%, albeit with limited sensitivity (42.1%). For the CSF-to-blood glucose ratio, the optimal cutoff was ≤ 0.25, which provided a more balanced profile with a sensitivity of 59.2%, specificity of 88.0%, and a PPV of 78.9%(Fig. 10).

Figure 9 Comparison of Diagnostic Accuracy for Different Glucose-Related Indicators. The combination of a low CSF glucose level (using either < 2.20 mmol/L or < 1.89 mmol/L threshold) with a low CSF-to-blood glucose ratio (≤0.4) achieved the highest diagnostic accuracy (AUC = 0.686), outperforming any of these individual parameters used alone.

### 3.5. Comparative performance of integrated diagnostic models

**Figure 11.**
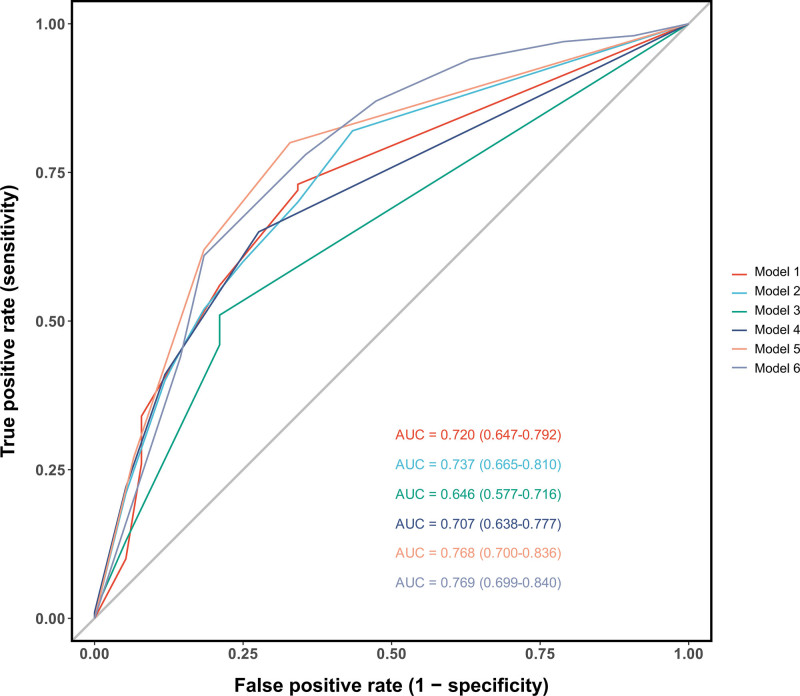
ROC analysis of 6 diagnostic models for CNS bacterial infection. Model 5 (integrating fever and stringent CSF criteria) and Model 6 (based solely on clinical criteria:T > 39.0°C, altered mental status, and intermittent fever) demonstrated the highest and comparable diagnostic accuracy (AUCs of 0.768 and 0.769, respectively). The performance of Model 6 highlights that a severe clinical phenotype can effectively identify infection without dependency on CSF laboratory results. AUCs = area under the curves, CNS = central nervous system, CSF = cerebrospinal fluid, ROC = receiver operating characteristic.

The diagnostic performance of 6 integrated models combining clinical and cerebrospinal fluid parameters was evaluated using ROC analysis (Fig. 11). The models and their respective AUC values were as follows:Model 1 (T > 38.0°C + CSF WBC ≥ 100 × 10^6^/L + [CSF glucose < 2.2 mmol/L OR CSF/Blood glucose ratio < 0.4]), AUC = 0..720 (95%CI: 0.647–0.792); Model 2 (T > 38.0°C + CSF WBC ≥ 1000 × 10^6^/L + the same glucose criteria), AUC = 0..737 (0.665–0.810); Model 3 (T > 38.0°C + [CSF WBC ≥ 100 × 10^6^/L OR the glucose criteria]), AUC = 0..646 (0.577–0.716); Model 4 (T > 38.0°C + [CSF WBC ≥ 1000 × 10^6^/L OR the glucose criteria]), AUC = 0..707 (0.638–0.777); Model 5 (T > 38.0°C + [CSF WBC ≥ 2000 × 10^6^/L OR the glucose criteria]), AUC = 0..768 (0.700–0.836); and Model 6 (T > 39.0°C + Altered Mental Status + Intermittent Fever), AUC = 0..769 (0.699–0.840). Notably, Model 6 achieved a high diagnostic accuracy comparable to the best laboratory-integrated model (Model 5) but through a distinct and clinically significant approach. Unlike Models 1 to 5, which combine fever with CSF laboratory abnormalities, Model 6 relies exclusively on 3 clinical parameters: high-grade fever (T > 39.0°C), altered mental status, and an intermittent fever pattern. This composition offers a unique advantage for rapid clinical triage, as it can raise a strong suspicion of CNS bacterial infection based solely on bedside assessment, potentially before the results of lumbar puncture and CSF analysis become available. Its high performance underscores the significant diagnostic value of this specific severe clinical phenotype and provides a quantitative validation of core clinical reasoning, which may be particularly valuable in resource-constrained settings.

## 4. Discussion

In this retrospective study of 176 patients suspected of PNBI of CNS, we developed and validated 6 diagnostic models. Our key findings are: (1) A model combining high-grade fever, altered mental status, and intermittent fever (Model 6) achieved high accuracy (AUC = 0.769) using clinical signs alone.(2) A model incorporating fever and stringent CSF criteria (Model 5) performed equally well (AUC = 0.768). (3) Altered mental status was the single most predictive clinical sign. (4) Extreme values of body temperature (>39.8°C) and CSF glucose (<1.01 mmol/L) offered high specificity for ruling in infection. (5) mNGS demonstrated superior pathogen detection compared to culture.

### 4.1. Methodological considerations and potential biases

To clarify the representativeness of our study population, we compared baseline characteristics between patients who underwent mNGS testing and the broader postoperative febrile population (Table 2), quantifying potential differences. Several methodological considerations should be noted. First, the high rate of antibiotic exposure before CSF collection (61.8%in the infection group) is a significant characteristic of our cohort. While this reflects real-world clinical practice, it may reduce the sensitivity of bacterial culture, potentially leading to misclassification of some true infections as noninfectious due to negative culture results, thereby affecting the apparent performance of our predictors. Second, although we included key variables in our final model, unmeasured confounding factors such as disease severity, surgical complexity, and immune status may still have influences. Finally, although we documented the use of invasive devices such as external ventricular drains, we did not analyze known infection risk factors including their indwelling time and ICU length of stay. Despite these limitations, our findings remain clinically valuable and informative for decision-making in this challenging clinical scenario.

### 4.2. Diagnostic value of fever and monitoring recommendations

Our findings confirm that fever is a nonspecific indicator following neurosurgery. The observation that over half (56.8%) of febrile patients in our cohort had no bacterial infection underscores the urgent need for better predictive tools to guide antibiotic stewardship and ensure prompt identification of true infections. As shown in Figure 2, the predictive accuracy of fever patterns – whether defined as a single temperature > 38°C or sustained fever > 38°C – was limited. ROC comparison suggested that intermittent fever had the highest AUC among patterns, though this finding should be interpreted with caution due to methodological constraints, The retrospective design and dynamic nature of temperature monitoring – where frequency was adjusted based on clinical status rather than fixed intervals – may have introduced heterogeneity in fever documentation. Stable patients on routine schedules may have had undocumented febrile episodes between measurements, while antipyretic interventions likely masked high or sustained fevers, leading to over representation of low-grade or intermittent fever in medical records. Notably, the cutoff analysis (Fig. 5) revealed that a temperature > 39.8°C was highly specific (99%) for bacterial infection. Therefore, in patients with postoperative fever and suspected intracranial infection, we recommend more frequent temperature monitoring and careful interpretation of fever patterns – taking into account any antipyretic interventions – to avoid missing significant fever patterns and enable early detection of sustained or high-grade fever.

### 4.3. Altered mental status:mechanisms and superior predictive value

Our findings (Fig. 6) indicate that decreased level of consciousness or altered mental status demonstrates higher predictive value (AUC) than body temperature for intracranial bacterial infection. Consciousness and mental status are fundamental brain functions, and their alterations directly reflect cerebral physiological changes. Evidence suggests that consciousness arises from neuron-glial interactions,^[17]^ while microglia – a key component of CNS immunity – play crucial roles in neuroinflammation.^[18]^ Bacterial infections induce cytotoxic effects and immune activation that disrupt neuronal and glial function, leading to neuronal injury and synaptic loss, which directly impair the physiological basis of cognition and mental status.^[19]^ Concurrently, infection can compromise blood-brain barrier integrity in the hippocampus, accelerating neurodegeneration and cognitive decline. Notably, infections may cause long-term structural changes;some patients exhibit cortical thickening nearly 2 years postinfection, significantly correlated with persistent cognitive sequelae. Thus, decreased consciousness or altered mental status represents a highly sensitive and specific indicator of intracranial bacterial infection in post-neurosurgical patients.^[3,20]^ A comparative study between community-acquired and post-neurosurgical bacterial meningitis confirmed that impaired consciousness (81%) and fever (83%) are the most common symptoms, substantially more frequent than headache (46.4%) or neck stiffness (42.5%).^[21]^ However, potential biases in our data should be acknowledged. Retrospective data capture – dependent on intermittent assessments by different clinicians – may fail to capture dynamic neurological changes. Patients with severe baseline neurological impairment may show limited observable deterioration, potentially increasing false negatives. Conversely, central fever or progression of primary neurological conditions may cause altered mental status unrelated to infection, elevating false positives. Baseline comparisons support this concern:patients with altered mental status had higher rates of hypertensive cerebral hemorrhage, external ventricular or lumbar drainage, and preoperative antibiotic prophylaxis. These differences suggest that“altered mental status”may be significantly influenced by primary neurological conditions and their interventions. Therefore, when PNBI of CNS is suspected, we recommend more frequent neurological assessments to accurately detect or rule out objective changes in mental status.

### 4.4. Reevaluation of CSF parameters: WBC count and glucose

Our study confirms that while elevated CSF WBC count is highly prevalent in post-neurosurgical patients (81.0%of noninfected patients had ≥ 100 × 10^6^/L), limiting its discriminatory value for PNBI, this finding partially aligns with Forgacs et al., who noted overlapping CSF profiles between chemical and bacterial meningitis.^[22]^ However, our results further refine this overlap by demonstrating that increasing the threshold to ≥ 2000 × 10^6^/L significantly improves diagnostic specificity, establishing it as a more convincing indicator for distinguishing PNBI of CNS (*P* < .001;Figure 6). This provides a crucial clue for identifying true infections amid the high-background“noise”of postoperative inflammation. Regarding CSF glucose, our findings diverge from the other view that considers < 1.89 mmol/L as a diagnostic threshold for bacterial meningitis.^[23]^ In our cohort, this cutoff demonstrated only moderate diagnostic performance (AUC = 0.651), with no significant advantage over the < 2.20 mmol/L threshold. This discrepancy may be attributed to unique confounders in the post-neurosurgical setting, such as CSF dilution from intraoperative saline irrigation and accelerated glucose metabolism due to surgical trauma and hemorrhage, potentially causing”pseudohypoglycorrhachia”in noninfected patients and thereby reducing the specificity of this marker. Although the CSF-to-blood glucose ratio ≤ 0.4 slightly improved the AUC, the difference remained nonsignificant, indicating limitations in relying solely on absolute or relative glucose values in postoperative patients. Nevertheless, the presence of profoundly low glucose levels (e.g.,<1.01 mmol/L, specificity 97%) should still raise strong suspicion of bacterial infection, suggesting a threshold-dependent diagnostic utility for this parameter.

### 4.5. Model comparison and clinical utility

Single clinical indicators – such as fever, decreased level of consciousness, or specific CSF parameters – often suffer from insufficient specificity or limited sensitivity in diagnosing intracranial infection after neurosurgery. This is primarily due to confounding influences from surgical trauma, underlying neurological diseases, and other noninfectious inflammatory responses. To overcome this limitation, this study integrated multiple indicators to develop 6 diagnostic prediction models. ROC analysis revealed that Model 5 (T > 38.0°C + [CSF WBC ≥ 2000 × 10^6^/L OR (CSF glucose < 2.2 mmol/L OR CSF/Blood glucose ratio < 0.4)]) demonstrated excellent discriminative ability, with an AUC of 0.768. Meanwhile, Model 6 (T > 39.0°C + Altered Mental Status + Intermittent Fever), which relies solely on clinical indicators, achieved a comparable AUC of 0.769, highlighting its potential for rapid identification without depending on CSF laboratory results. Compared to previously reported prediction models^[24,25]^ – such as the one by Jun Nie et al., which incorporated 8 variables and achieved an AUC of 0.90 in the validation set, or Tiantian Zhai model, which attained 85%sensitivity and 100%specificity – the best-performing model in our study yielded a slightly lower AUC of 0.768. However, our model was specifically designed to address the high heterogeneity of the post-neurosurgical population and aimed to correct for the“background noise”induced by the surgery itself. Notably, Model 6, which utilizes only 3 bedside-accessible parameters, offers a practical solution for initial screening in settings with limited diagnostic resources or when rapid assessment is required. It is important to note that while Model 5 holds a slight advantage in overall diagnostic performance, Model 6 provides unique value for dynamic monitoring and early warning due to its completely noninvasive nature and ease of repeated assessment. These 2 models complement each other, expanding the range of diagnostic options for different clinical scenarios. In summary, the diagnostic algorithms developed in this study, particularly Models 5 and 6, provide specialized tools for discriminating CNS infections following neurosurgery. They not only validate the utility of a multi-parameter integration strategy in improving diagnostic accuracy but also emphasize the importance of incorporating clinical practicality and population-specific characteristics into model development. Future research should build upon this foundation, utilizing prospective data to further refine variable definitions and data collection processes, thereby enhancing the models’generalizability and precision in clinical application.

### 4.6. Regarding the diagnostic gold standard

Although this study employed CSF mNGScombined with bacterial culture and clinical treatment response as the diagnostic gold standard, this approach has inherent limitations. First, mNGS testing was not performed on all post-neurosurgical patients with fever but was conducted based on clinical suspicion and patient consent, potentially introducing selection bias. Our cohort may therefore overrepresent patients with more severe or complex clinical presentations, which could limit the generalizability of our findings to the broader population of postoperative febrile patients. Second, the unpredictable timing of infection onset, combined with uncertainties in clinical decision-making and the process of obtaining family consent for CSF sampling, means that not all mNGS and culture specimens were collected at the initial stage of infection. In standard clinical practice, when intracranial bacterial infection is suspected, lumbar puncture is typically performed to collect CSF for routine and biochemical analysis, often accompanied by bacterial culture and sometimes the placement of external lumbar drainage. If CSF biochemical results suggest a high probability of infection, antimicrobial therapy is initiated immediately, with follow-up CSF biochemical tests and mNGS performed 1 to 3 days later. After drainage and antibiotic treatment, CSF biochemical parameters may have normalized and mNGS results may turn negative, even though the patient is still clinically diagnosed with an infection based on comprehensive assessment. Furthermore, in some bacterial infections (such as Acinetobacter baumannii), despite clinical improvement after antimicrobial therapy and CSF drainage – including resolution of fever, stabilization of mental status, and decreased CSF WBC counts – mNGS may still detect bacterial presence due to the pathogen’s strong environmental adaptability and persistence [Insert reference]. These factors can create significant discrepancies at any single time point between the clinical presentation, CSF parameters, and the final gold-standard diagnosis, thereby introducing bias into statistical conclusions. To obtain more reliable evidence, future prospective studies should establish unified definitions and standardized documentation for key diagnostic indicators. We recommend that specially trained research personnel be responsible for data collection, with particular attention to the following: for patients with high-risk factors for infection (e.g., CSF leakage), baseline body temperature should be established preoperatively or in the early postoperative period, with increased monitoring frequency; the recording time of all clinical indicators (e.g., body temperature, mental status, nuchal rigidity) and laboratory parameters (e.g., complete blood count, blood glucose) should be strictly synchronized with CSF sampling;additionally, specimens for CSF routine biochemistry, bacterial culture, and mNGS should be aliquoted from the same puncture sample to minimize inter-sample variability and ensure result consistency.

### 4.7. Study limitations

This study has several inherent limitations, as discussed in the preceding sections. First, its retrospective, single-center nature may limit generalizability, and the selective use of mNGS based on clinical suspicion could have introduced spectrum bias. Second, the high prevalence of pretest antibiotic exposure – a key characteristic of our cohort – represents a major confounding factor, as noted earlier. Third, the definitions of key clinical predictors were subject to the variability of real-world clinical practice rather than a standardized protocol. Finally, as an initial model development study, it requires external validation to confirm its utility.

## 5. Conclusion

This study establishes a stepwise diagnostic strategy based on objective indicators to address the challenge of discriminating intracranial bacterial infection following neurosurgery. Our results demonstrate that combining the highly sensitive clinical alert of“high fever with altered mental status”with the highly specific criterion of”elevated CSF parameters (e.g., WBC ≥ 2000 × 10^6^/L) or profoundly low glucose levels”enables effective risk stratification prior to CSF pathogen testing. Although the retrospective design, with its variability in body temperature monitoring frequency, and the restriction of the study population to those who underwent mNGS testing may affect the generalizability of the findings, several measures were taken to maximize reliability. These included the adoption of a composite gold standard (mNGS combined with culture and clinical treatment response) and the inclusion of a substantial sample size from a high-volume neurosurgical center, thereby strengthening diagnostic classification and data analysis. In summary, this study confirms that moving beyond reliance on single parameters toward a systematic, combined evaluation is crucial for accurately identifying infection and optimizing antibiotic stewardship. Future prospective, multicenter studies are warranted to further validate and refine this diagnostic pathway.

## Author contributions

**Conceptualization:** Baoli Lin, Xianbing Meng, Tao Fu.

**Data curation:** Xianbing Meng, Nanyue Peng.

**Formal analysis:** Baoli Lin.

**Funding acquisition:** Qingguo Li.

**Investigation:** Baoli Lin, Tao Fu, Nanyue Peng.

**Methodology:** Baoli Lin, Xianbing Meng.

**Project administration:** Baoli Lin, Xianbing Meng, Qingguo Li.

**Resources:** Qingguo Li.

**Software:** Baoli Lin, Xianbing Meng.

**Supervision:** Ke Pu, Qingguo Li.

**Validation:** Qingguo Li.

**Visualization:** Tao Fu, Qingguo Li.

**Writing – original draft:** Baoli Lin.

**Writing – review & editing:** Xianbing Meng.

## References

[1] Hernandez Ortiz OH Garcia Garcia HI Munoz Ramirez F. Development of a prediction rule for diagnosing postoperative meningitis: a cross-sectional study. J Neurosurg. 2018;128:262–71. 28298047 10.3171/2016.10.JNS16379

[2] McClelland SR Hall WA. Postoperative central nervous system infection: incidence and associated factors in 2111 neurosurgical procedures. Clin Infect Dis. 2007;45:55–9. 17554701 10.1086/518580

[3] Chojak R Kozba-Gosztyla M Gaik M. Meningitis after elective intracranial surgery: a systematic review and meta-analysis of prevalence. Eur J Med Res. 2023;28:184. 37291583 10.1186/s40001-023-01141-3 PMC10249328

[4] Sajjad J Kaliaperumal C Yousaf I Bhatti RJ O’Sullivan M. A prospective analysis of complications of intracranial tumor surgery. J Neurol Surg A Cent Eur Neurosurg. 2017;78:53–9. 27415593 10.1055/s-0036-1584814

[5] Kar M Jamwal A Dubey A Sahu C Patel SS. Bacterial meningitis among intracranial surgery patients at a university hospital in Northern India. Indian J Crit Care Med. 2022;26:1244–52. 36755630 10.5005/jp-journals-10071-24363 PMC9886024

[6] Al Yazidi LS McMullan B Kohan S Palasanthiran P. Persistent gram-negative neurosurgical meningitis in a neonate, successfully treated with intraventricular colistin: case report and review of the literature. Pediatr Infect Dis J. 2018;37:e79–81. 28841583 10.1097/INF.0000000000001743

[7] Yue Z Zhi X Bi L Zhao L Ji J. Treatment and prognostic risk factors for intracranial infection after craniocerebral surgery. Neurosurg Rev. 2023;46:199. 37568062 10.1007/s10143-023-02106-0

[8] Scheithauer S Burgel U Bickenbach J. External ventricular and lumbar drainage-associated meningoventriculitis: prospective analysis of time-dependent infection rates and risk factor analysis. Infection. 2010;38:205–9. 20333433 10.1007/s15010-010-0006-3

[9] Bakhshi SK Suhail N Mitha R Moazzam M Zahid N Shamim MS. Lumbar drain for temporary cerebrospinal fluid diversion: factors related to the risks of complications at a university hospital. World Neurosurg. 2020;143:e193–8. 32711138 10.1016/j.wneu.2020.07.120

[10] Hocker SE Tian L Li G Steckelberg JM Mandrekar JN Rabinstein AA. Indicators of central fever in the neurologic intensive care unit. JAMA Neurol. 2013;70:1499–504. 24100963 10.1001/jamaneurol.2013.4354

[11] Horan TC Andrus M Dudeck MA. CDC/NHSN surveillance definition of health care-associated infection and criteria for specific types of infections in the acute care setting. Am J Infect Control. 2008;36:309–32. 18538699 10.1016/j.ajic.2008.03.002

[12] Neuroimmunology Group of Neurology Branch of Chinese Medical Association, Neuroimmunology Committee of Chinese Society for Immunology, Immunology Society of Chinese Stroke Association. Chinese expert consensus on the diagnosis and treatment of central nervous system infection in neurosurgery. Chin. J. Neurosurg. 2021;37:2–15.

[13] Staal SL Olie SE Ter Horst L. Granulocytes in cerebrospinal fluid of adults suspected of a central nervous system infection: a prospective study of diagnostic accuracy. Infection. 2024;52:1415–23. 38520645 10.1007/s15010-024-02200-5 PMC11289325

[14] Mikulska M Furfaro E Del Bono V. (1-3)-beta-D-glucan in cerebrospinal fluid is useful for the diagnosis of central nervous system fungal infections. Clin Infect Dis. 2013;56:1511–2. 23392391 10.1093/cid/cit073

[15] Tunkel AR Hasbun R Bhimraj A. 2017 infectious diseases society of America’s clinical practice guidelines for healthcare-associated ventriculitis and meningitis. Clin Infect Dis. 2017;64:e34–65. 28203777 10.1093/cid/ciw861 PMC5848239

[16] Raviv N Field N Adamo MA. Postoperative fever workup in pediatric neurosurgery patients. J Neurosurg Pediatr. 2020;26:691–5. 32947257 10.3171/2020.5.PEDS2019

[17] Robertson JM. The gliocentric brain. Int J Mol Sci. 2018;19 :3033. 30301132 10.3390/ijms19103033 PMC6212929

[18] Klein RS Hunter CA. Protective and pathological immunity during central nervous system infections. Immunity. 2017;46:891–909. 28636958 10.1016/j.immuni.2017.06.012 PMC5662000

[19] Zhang H Jin Q Li J. Astrocyte-derived complement C3 facilitated microglial phagocytosis of synapses in Staphylococcus aureus-associated neurocognitive deficits. PLoS Pathog. 2025;21:e1013126. 40294039 10.1371/journal.ppat.1013126 PMC12121917

[20] van de Beek D de Gans J Spanjaard L Weisfelt M Reitsma JB Vermeulen M. Clinical features and prognostic factors in adults with bacterial meningitis. N Engl J Med. 2004;351:1849–59. 15509818 10.1056/NEJMoa040845

[21] Parlayan HNK Solay AH Aksoy BR Bulut D Hojabri M Sencan I. Clinical insights and outcomes in community-acquired acute bacterial meningitis <em>versus</em> postoperative bacterial meningitis. J Coll Physicians Surg Pak. 2024;34:1441–7. 39648377 10.29271/jcpsp.2024.12.1441

[22] Forgacs P Geyer CA Freidberg SR. Characterization of chemical meningitis after neurological surgery. Clin Infect Dis. 2001;32:179–85. 11170905 10.1086/318471

[23] Shahan B Choi EY Nieves G. Cerebrospinal fluid analysis. Am Fam Physician. 2021;103:422–8. 33788511

[24] Nie J Zhang W Zhang H. Development and validation of a predictive model for postoperative intracranial infections in neurosurgery with risk factor analysis. World Neurosurg. 2024;189:e126–40. 38857869 10.1016/j.wneu.2024.05.184

[25] Zhai T Fu ZL Qiu YB Chen Q Luo D Chen K. Application of combined cerebrospinal fluid physicochemical parameters to detect intracranial infection in neurosurgery patients. BMC Neurol. 2020;20:213. 32460716 10.1186/s12883-020-01781-6 PMC7251726

